# Performance Evaluation of the Radio Propagation in a Vessel Cabin Using LoRa Bands

**DOI:** 10.3390/s26010207

**Published:** 2025-12-28

**Authors:** Kun Yang, Zebo Shi, Li Qin, Jinglong Lin, Chen Li

**Affiliations:** 1 Department of Information Engineering, Zhejiang Ocean University, Zhoushan 315022, China; yangkun@zjou.edu.cn (K.Y.); lingjinglong@zjou.edu.cn (J.L.); 2 Ocean Connectivity Laboratory, Zhejiang Ocean University, Zhoushan 315022, China; shizebo@zjou.edu.cn; 3 Department of Naval Architecture and Maritime, Zhejiang Ocean University, Zhoushan 315022, China; 4 Zhejiang Intertion Information Technology Co., Ltd., Zhoushan 316000, China; lichen@intertion.net

**Keywords:** LoRa, channel measurement, RSS, SINR

## Abstract

Due to the development of the Internet of Things (IoT) and maritime wireless networks, the wireless networking of vessels will be the future trend. Furthermore, long-range (LoRa) technology is widely used in the marine field with the benefits of long range, lower power consumption, security, scalability, and robustness. In this study, LoRa is used as the solution for internal wireless networks of vessels as well as considering external and internal wireless communication, aiming to reduce construction and maintenance costs. The received signal strength (RSS) and signal to interference plus noise ratio (SINR) were measured and analyzed. The findings demonstrated that the mean value of the RSS and the SINR in the cockpit are above −81.70 dBm and 4.45 dB respectively, which indicates that there is a good communication link between the deck and the cockpit. Furthermore, the RSS value acquired by the nodes located on the same side of the gateway is stronger than that of the other nodes. Additionally, the RSS value acquired by the nodes close to the windows is found to be as high as 6–9 dB over that of the node located in the middle of the cockpit.

## 1. Introduction

### 1.1. Background of This Study

Traditional vessel area networks and data transmission are mainly built on wired networks, which can bring about many problems, such as high wiring costs, drilling holes for wiring, and difficulty in maintenance [1,2]. With the development of the IoT, wireless technology will become the direction of future development in the field of vessels [3,4]. Several studies verified the feasibility of LoRa for long-distance vessel-to-vessel and vessel-to-shore communication [5,6]. If LoRa can be applied to the domain of intra-vessel wireless communication, it will reduce the number of network devices that need to be deployed and thus reduce costs.

Unlike aircraft cabins and factories with multipath metal environments, vessel cabins have water-tightness requirements, especially for special vessels such as oil tankers, chemical tankers, warships, etc. Therefore, traditional channel characteristics and modeling methods are unsuitable for vessel environments [7]. In a watertight environment, signals will mostly propagate through steel plates. Thus, in-depth research is needed on the propagation characteristics and models of signals on multiple decks. This study will test the possibility of signal propagation using LoRa bands in vessels’ cabin and main deck environments, with the aim of providing suggestions and guidance on deploying internal networks in vessels.

Furthermore, RSS and SINR are crucial metrics for evaluating the effectiveness of communication systems, particularly in wireless communication, as they directly impact the quality and dependability of communication [8]. The packet loss rate of LoRa technology can usually be controlled at a very low level thanks to its spread spectrum technology, forward error correction coding, adaptive data rate adjustment, and optimized network design. These characteristics ensure that LoRa can provide stable communication connections in various environments. Therefore, RSS and SINR were used to test the reliability of the communication in this study.

### 1.2. Related Works

With the development of the IoT and ocean communication, more and more wireless ocean devices are appearing on the sea surface, such as autonomous underwater vehicles (AUVs), unmanned surface vehicles (USVs), unmanned aerial vehicles (UAVs), and autonomous vessels.

Due to LoRa’s low power consumption and long transmission distance, it plays an increasing role in long-distance ocean communication [9]. Moreover, significant efforts have been made on this compelling technology since its emergence. LoRa performs well in long-distance ocean communication, and it was verified that links are feasible within 22 km [10]. A wireless sensor system prototype was constructed as a solution for Salton Sea environmental monitoring, using readily available commercial components to collect on-site sensor data from the Salton Sea and Empire Valley regions [11]. The performance of LoRa technology in developing monitoring systems was explored [12], embedding a microcontroller into LoRa radio shielding and combining it with multiple sensors as a sensor node to send data to a remote gateway. In terms of aquaculture, a transmission channel had been established from an offshore monitoring structure equipped with long-range wide-area network (LoRaWAN) transmitters to an onshore receiving device consisting of two LoRaWAN gateways, which can achieve effective data transmission within at least 8.33 km [13]. A test was conducted using a passenger vessel and a research vessel as endpoint nodes [14] and gateways located in inland mountainous and hilly areas. The results indicated that LoRa can transmit over 110 km in free-space scenarios and over 20 km in coastal urban environments. In addition, LoRa has played a significant role in monitoring fish populations in the fishing industry [15,16].

When it comes to the ocean, vessels cannot be ignored. Unmanned vessels, or autonomous vessels [17,18], will be the main direction of future vessel development. There have been many studies in vessel data monitoring applications, first using various sensors and multi-hop networks to collect vessel data [19,20,21]. The feasibility of wireless sensor networks onboard vessels has been successfully verified through a large-scale ship-borne wireless sensor network architecture based on layered regions [22]. With the development of LoRa, it is gradually being used in vessel monitoring, with applications for transmitting sailboat data and vessel temperature data [23,24].

However, due to the unique steel multipath environment of vessels and the water-tightness requirements of some vessels, deploying networks onboard vessels previously required multiple uses of repeaters, resulting in increased costs. Therefore, it is necessary to study the characteristics of wireless signals in vessel cabin environments. After verification, it has been found that establishing a wireless vessel network directly in the unique environment of the cabin is challenging. Previous studies on traditional wireless technologies in vessel cabins have suggested that, due to the harsh metallic multipath environment, repeaters might be recommended to improve network quality even in LOS scenarios [25,26,27,28,29]. Ray tracing has been used multiple times in vessel cabins to establish a channel model for the cabin environment [30,31]. However, due to the vessel’s motion and the onboard equipment’s noise and vibration, there is a gap between the established model and reality. Meanwhile, to improve the transmission quality of signals, MIMO and OFDM are both used in vessels [32,33,34,35]. In addition, the impact of personnel in the cabin on signals has also been studied [36].

Of course, with the use of LoRa on vessels, research on LoRa channels has also been put on the agenda, first in general indoor environments [37] and secondly in modeling vessel engine room environments using ray tracing methods [38,39]. However, a single-engine room environment can be considered the same indoor environment. Due to the special water-tightness requirements of vessels, it is necessary to use LoRa for testing and research in different compartments. This study aims to fill the gap in this area.

In summary, significant progress has been made in the above areas in existing re-search. However, through the literature review, it can be found that the following research shortcomings exist: most studies focus on the propagation characteristics within a single compartment (such as the engine room) or the same deck. For complex ship environments with strict water-tightness requirements, composed of multiple steel decks and compartments, there is still a lack of systematic empirical research, especially regarding the penetration performance of signals between different decks. In the complex multipath and dynamic environment of real sailing ships, the work of measuring and analyzing LoRa link performance (such as RSS and SINR) is still limited.

Therefore, the work of this article is aimed at addressing the aforementioned shortcomings. This study aims to systematically evaluate the propagation performance of LoRa in ship cabin environments spanning multiple decks through a measurement activity conducted on a real research vessel, with a focus on path loss, RSS, and SINR, in order to provide an empirical basis and specific deployment guidelines for deploying reliable LoRaWAN networks in such complex and special environments.

### 1.3. Novelty of This Study

The main contributions and novelty of this article are summarized as follows:

Scene uniqueness: This study focuses on the special steel ship cabin environment with water-tightness requirements, which is significantly different from ordinary indoor or multi-metal factory environments. We conducted empirical measurements on a real re-search vessel to address this unique and less studied communication scenario.

Cross-deck propagation analysis: We systematically measured and analyzed the propagation performance of LoRa signals between different decks of the ship (from the top deck to the bridge and main deck), particularly evaluating the path loss, RSS, and SINR of signals penetrating steel decks and watertight structures, which are relatively rare in the existing literature.

Deployment guidance suggestion: Based on measured data, we quantitatively analyzed for the first time the relative orientation between nodes and gateways, as well as the significant impact of proximity to windows on signal strength in LoRa deployment on ships. Based on this, we proposed specific, data-supported network node deployment guidelines.

Model validation and electromagnetic environment assessment: We compared and validated the measured path loss with the standard channel model, and revealed the important feature of maintaining stable electromagnetic interference levels in ship cabins in the LoRa frequency band through Pearson correlation analysis of RSS and SINR.

The rest of the paper is organized as follows: Section 2 briefly introduces the methodology and the experimental details. Section 3 presents the analysis of the signal data for the cockpit and the main deck and compares the signal data of the cockpit and the main deck. Finally, the conclusions are drawn in Section 4.

## 2. Materials and Methods

### 2.1. Experimental Set-Up

**Figure 1:**
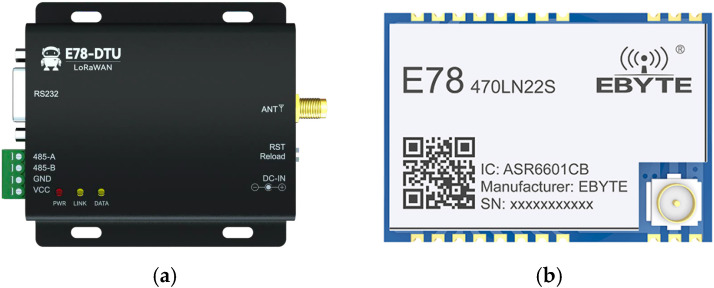
Connection of experimental hardware. (**a**) E78-DTU. (**b**) E78-470LN22S LoRaWAN Module.

This test uses E78-DTU (Ebyte, Chengdu, China) (Figure 1a) as the testing equipment, which is a standard LoRaWAN node radio developed based on the E78-470LN22S (Ebyte, Chengdu, China) (Figure 1b) wireless module and operates in the frequency range of CN470~510 MHz, supports CLASS-A/CLASS-C node types, and supports both Activation by Personalization (ABP) and Over-The-Air Activation (OTAA) network access methods.

LoRa is a low-power local area network wireless standard developed by Semtech-based on a spread spectrum modulation technology called chirp spread spectrum (CSS) [40,41]. The performance of a LoRa link can be configured by adjusting several key parameters, which involves making trade-offs among communication range, data rate, and power consumption [8,14], as follows:

Bandwidth (BW) represents the bandwidth of the radio signal. The available BW settings in LoRa are 125 kHz, 250 kHz, and 500 kHz.

Spreading factor (SF) represents the number of bits encoded per chirp, ranging from 7 to 12. For example, SF10 means that every chirp represents ten bits. Increasing the SF improves the link budget and enhances interference resistance, but it also reduces the data rate and increases the on-air time, leading to higher power consumption.

Coding rate (CR) controls the amount of forward error correction (FEC) redundancy added to the data. It is denoted 4/(4 + *n*), where n is an integer from 1 to 4, corresponding to the CR values 1 through 4, respectively. This results in code rates of 4/5, 4/6, 4/7, and 4/8. A lower code rate (e.g., 4/8 corresponding to CR = 4) introduces more redundancy, which enhances the robustness against interference and forward error correction capability at the expense of a reduced effective data rate. Conversely, a higher code rate (e.g., 4/5 corresponding to CR = 1) offers less protection but a higher data throughput.

LoRaWAN is the media access control (MAC) layer communication protocol based on the LoRa technology, designated for low-power and low-data-rate communication purposes that can be used directly to communicate with the gateway.

**Figure 2:**
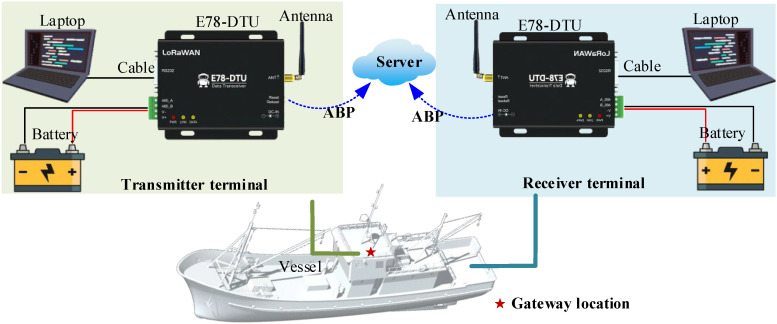
Connection of experimental hardware.

As shown in Figure 2, one E78-DTU module is placed at the top as a gateway and the other as a receiver placed in the cabin or main deck. The external communication interface of the data transmission radio adopts RS485 interface communication, and users can connect to the standard LoRaWAN network through AT commands or simple configuration on the upper computer. The ABP model is adopted in the experiment because it streamlines the process, enabling rapid validation and offering exceptional debugging reliability.

### 2.2. Experimental Parameters

Prior to testing, the device was calibrated, and the serial port debugging assistant was initiated. The serial port baud rate was set to 9600 bps with an 8N1 verification method, and network access parameters were configured via AT commands. Before deployment, all experimental equipment underwent functional validation in clear, unobstructed outdoor conditions. The measured path loss at 50 m under free-space conditions was compared with the theoretical free-space path loss (FSPL) formula. The calculated FSPL was 59.96 dB, while the average measured path loss over 5 min was 60.72 dB, yielding a residual error of 0.76 dB (within acceptable limits) and a standard deviation (SD) of 0.05 dB, indicating stability. Thus, a 5 min testing duration was adopted for each measurement point. Point-to-point communication confirmed that all devices could reliably transmit and receive data packets under the specified power and parameter configurations.

The testing equipment is based on the STM32MP157C chipset and is outfitted with the SX1302 LoRaWAN gateway. It uses a standard Mini PCI-e form size and offers USB and Universal Asynchronous Receiver-Transmitter (UART) interfaces. A power amplifier (PA) and a low-noise amplifier (LNA) are both incorporated within the module, which also has a full-duplex design [39]. It can function as both the gateway and node modes, from which a dipole antenna with a gain of 3 dBi was installed at both the gateway and the node sides. The gateway transmission power is 22 dBm at the frequency range of 470 MHz and 510 MHz. Continuous data collection was conducted at each measurement node location. The sampling frequency was one sample recorded every 10 s. The RSS and SINR values analyzed subsequently were the statistical averages of all time samples at that location, in order to eliminate short-term channel fluctuations caused by slight ship shaking, personnel movement, and other factors. The experimental parameters are summarized in Table 1.

### 2.3. Experimental Campaign

**Figure 3:**
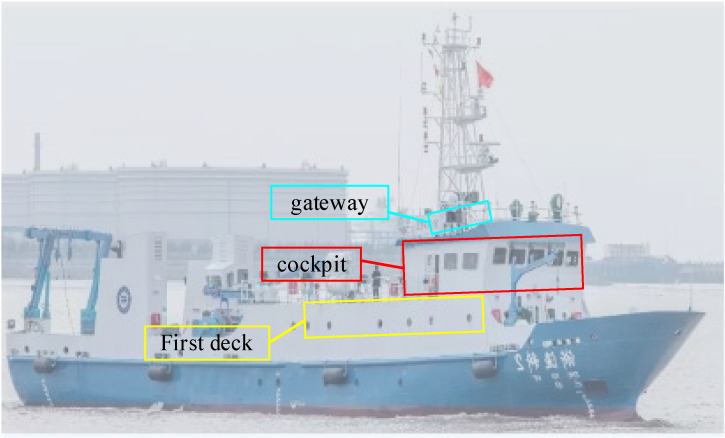
The placement of the gateway and the measurement nodes in this study.

**Figure 4:**
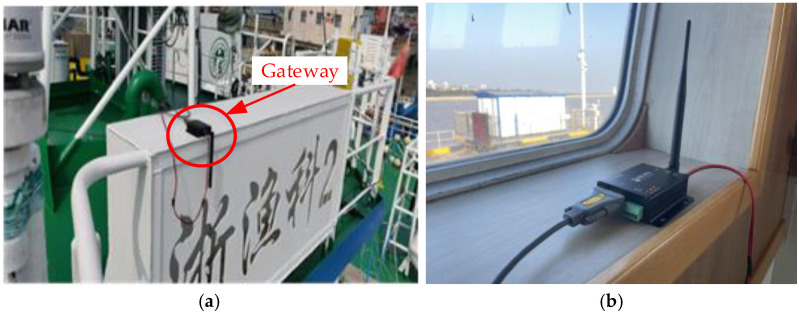
(**a**) The gateway location; (**b**) the measurement node location.

To study the RSS and SINR comprehensively in the vessel cabin scenario, a measurement campaign was carefully planned and conducted in a scientific research vessel (*Zheyuke 2*) in Zhoushan Port, China. The scientific research vessel was employed for marine fishery resource surveys, offshore teaching and internships, and multidisciplinary research in marine meteorology, chemistry, biology, geology, environmental studies, hydrology, and diving and drilling, which can be regarded as typical cabin scenarios. Additionally, the experimental vessel has a length of 48.8 m, a width of 8 m, a total tonnage of 290 tons, and a top speed of 12 knots. The test gateway and cabin locations are shown in Figure 3, and the physical images of the gateway and nodes are shown in Figure 4. This gateway deployment method (directly placed on the top deck) represents the most practical application scenario for onboard ship environments. The gateway’s outer casing is constructed from plastic, and its connections are sealed with waterproof tape, effectively preventing equipment corrosion.

In this test, we employed point-to-point sequential testing, ensuring all data were collected using the same hardware platform by conducting sequential measurement with a single node. Continuous measurements at a fixed location with this node allowed us to obtain stable, clean channel response data over time for the specified point.

**Figure 5:**
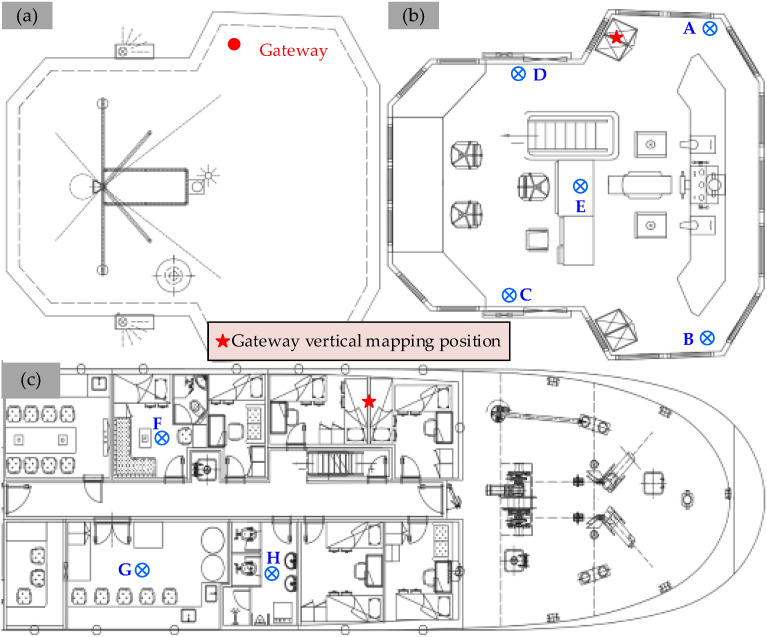
(**a**) Gateway location (top deck); (**b**) node locations (cockpit); (**c**) node locations (main deck).

During the experiment, a gateway was placed on the top deck of the experimental vessel with a LoRa antenna oriented downward, as shown in Figure 5a. The measurement node was placed on the five positions (A–E) of the cockpit and the three positions (F–H) of the main deck with the antenna oriented upward, as shown in Figure 5b and Figure 5c, respectively. The height between the cockpit and the main deck is around 2.35 m, and the hull is made of steel and wooden structures.

The gateway antenna is mounted on the top deck surface, with a metallic deck beneath it. This arrangement creates a reflective plane, altering the antenna’s actual radiation pattern compared to its free-space behavior. While this deployment configuration deviates from idealized conditions, it closely replicates real-world shipboard environments. One of the goals of this study is to evaluate the link performance under such practical deployment conditions.

During the measurements, the research vessel “*Zheyuke* 2” was stationary and docked at Zhoushan Port. This approach was taken to control variables, eliminating the additional and complex effects on the wireless signal caused by dynamic factors such as changes in the vessel’s attitude (e.g., rolling, pitching) and main engine vibrations during sailing. This allowed us to more purely investigate the impact of the vessel cabin’s static structure (e.g., steel decks, bulkheads) itself on signal propagation. In addition, the temperature during the experiment was 59 °F and the relative humidity was 30%.

## 3. Result and Discussion

### 3.1. Original Data

This study uses Java Script Object Notation (JSON) objects to code LoRaWAN messages. All items inside the Received Package (RXPK) (from the gateway to measurement nodes) object are reported in Table 2. RSS and SINR values used in the following analysis are extracted from the RXPK object.

### 3.2. Path Loss Analysis and Modelling

#### 3.2.1. Logarithmic Distance Path Loss Model Analysis

The path loss (PL) model characterizes the attenuation of signal power during the propagation through a medium. It accounts for phenomena including free-space wave expansion, as well as reflection, scattering, diffraction, and absorption. Path loss is influenced by environmental conditions, the distance between transmitter and receiver, and the height of the transmit and receive antennas [42].

Based on data from all eight measurement points, the logarithmic distance path loss model is modeled using the least squares method,

(1) $$PL(d)=PL(d_{0})+10n\log_{10}(\frac{d}{d_{0}})+X_{\sigma }$$

where *PL*(*d*) is the path loss, *d* is the Tx Rx distance, *PL*(*d*_0_) is the path loss at the reference distance *d*_0_, *n* is the path loss index, and *X_σ_* is a normally distributed random variable representing shadow fading (with a mean of 0 and a SD of *σ*).

The fitting results are as follows: the path loss *PL*(*d*_0_) of the model at the reference point is −73.96 dB, and the path loss index *n* = 7.7. Fitting goodness R^2^ = 0.87. In addition, the calculated SD of shadow fading is *σ* = 4.20 dB.

The residual between the measured and predicted values of the logarithmic loss model for each node shown in Table 3.

The extremely high path loss index (*n* = 7.7) quantitatively reveals the extreme challenges of the ship’s steel cabin environment. Compared to free-space (*n* = 2) or typical indoor environments (*n* = 3 or 4), the attenuation rate of signals with distance in this research scenario is much more severe. This is mainly attributed to the multiple reflections, diffractions, and significant energy losses caused by electromagnetic waves passing through steel decks in a sealed metal environment. A moderate SD of shadow fading (*σ* = 4.20 dB) indicates that at a specific distance, signal random fluctuations caused by factors such as metal equipment inside the cabin, door and window opening and closing, and personnel movement are within an acceptable range. This value is comparable to some complex indoor factory environments, and it suggests that when planning the network, we need to consider a link margin of about 4.20 dB to counteract this slow fading effect and ensure the reliability of the link.

#### 3.2.2. 3GPP InF-SL Model Analysis

Due to the similarity between the cabin environment and the factory environment, both have a main structure composed of metal (steel beams, cabin walls/factory walls, machines). These metal surfaces have high reflectivity and strong shielding properties against electromagnetic waves, which are the main reasons for severe multipath fading and penetration loss of signals. For this reason, this study characterizes the PL model according to the InF-SL model in the 3GPP.38.901 [43] which can be expressed as,

(2) $$PL=a+b\log_{10}(d)+c\log_{10}(f_{c})$$

where *a*, *b*, and *c* are constants derived from 3GPP TR 38.901 V17.0.0 (2022-06), specifically Table 7.4.1-1: Pathloss models; *d* is distance between Tx and Rx with an accuracy of approximately ±0.2 m; and *f_c_* denotes the center frequency.

In this study, due to the inherent physical characteristics of radio waves (where lower frequencies correspond to longer wavelengths and enhanced diffraction and penetration capabilities), we selected 475 MHz, a frequency close to 470 MHz and within the LoRa frequency band permitted in China. So, *f*_c_ is 475 MHz; thus the PL model can be expressed as

(3) $$PL=73.13+82.9\log_{10}(d)$$

The residual between the measured and predicted values of the InF-SL model for each node shown in Table 4.

**Figure 6:**
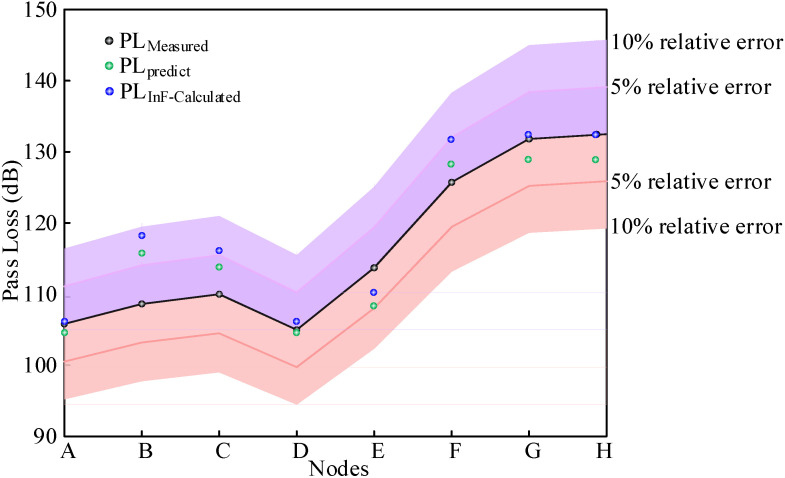
Measured and calculated path loss values.

Figure 6 illustrates the measured path loss values alongside the predicted values derived from Equations (1) and (3). A notable observation was that all calculated values fall within a 10% error margin relative to the measured values, with the majority residing within an even tighter 5% error range. This demonstrated that the calculation models exhibit high accuracy and effectively replicate the measured data.

### 3.3. RSS Analysis

It is important to study the difference in the measurement results between the cockpit and the main deck so that a better link budget can be planned in this scenario and the communication link limit can be predicted, which can be further used as crucial information for the mesh network planning.

**Figure 7:**
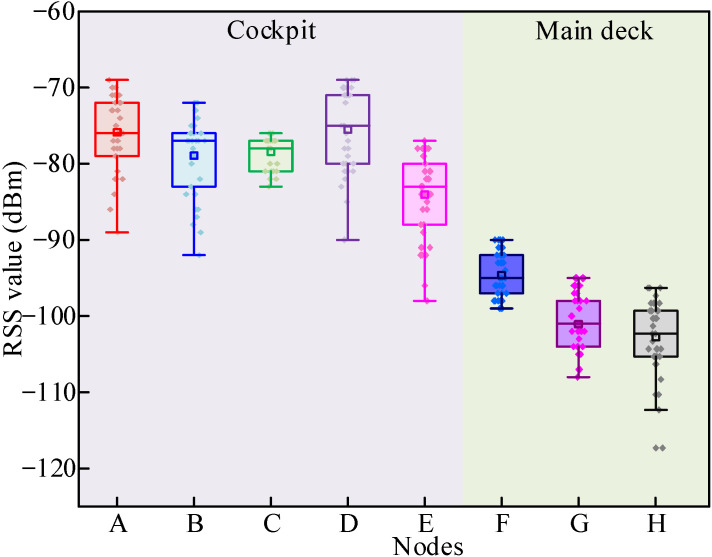
RSS values were acquired in the cockpit and the main deck.

Figure 7 shows the boxplot for the RSS value acquired by all nodes, which includes the mean, minimum, and maximum values. It can be seen that the RSS values acquired by nodes in the cockpit (A~E) are much higher than those by nodes on the main deck (F~H). This indicates the significant influence of the penetration loss between the decks. However, it needs to be pointed out that the difference in the RSS value between nodes on the same side of the cockpit, besides node E, is rather small (<2 dB), which indicates the location on the same side as the gateway is recommended for the node installation in the lower deck if the link quality is not good. Furthermore, it can be concluded that at the constant vertical receiving distance, nodes located on the same side as the gateway (A, D, and F) received higher RSS values than other nodes. Additionally, the RSS values acquired by the nodes adjacent to the windows (node A and D) are as high as 6–9 dB over the nodes positioned in the cockpit’s center. This observation supports the recommendation for node placement in this particular circumstance.

Table 5 presents the mean and SD values of RSS measured by all nodes. All average RSS calculations in this study adhere to the physical principle of power averaging. The SD value can be expressed as

(4) $$\sigma =\sqrt{\frac{1}{N-1}\sum_{i=1}^{N}({\mathrm{RSS}}_{i}-\bar{\mathrm{RSS}}{)}^{2}}$$

where *σ* denotes the SD of RSS in dB, *RSS_i_* represents the RSS measurement from node *i* (*i* = A, B, C, D, E, F, G, H) in dBm, and *N* is the total number of measurement nodes.

As observed in Table 5, the average RSS value acquired by the cockpit nodes ranges from −81.70 dBm to −72.97 dBm, while the average RSS value acquired in the main deck ranges from −100.49 dBm to −93.73 dBm. It can also be inferred from Table 5, node ABDE is located in the cab, and there may be dense metal equipment or structures (such as the cab and instrument cabinet) between it and the gateway, which constitute the main obstructions and result in additional penetration losses that the model did not take into account. The SD of RSS value is up to 5.53 dB, which may primarily be attributed to the vessel’s movement and the presence of multipath reflections in the indoor environment. Moreover, the results indicated that the link quality is satisfactory for transmitting data, even via a single deck that is made of steel and wooden structures. The RSS SD at most locations remained at a low to moderate level, indicating relatively stable signal strength over the 5 min observation window. However, nodes ABDE exhibited an SD twice that of node C under similar environmental conditions, highlighting the significant impact of multipath propagation and surroundings on signal variability, even among nodes in comparable settings. Additionally, nodes G and H, operating in critical signal reception zones, demonstrated extreme sensitivity to minor environmental fluctuation, resulting in notably high SD values. It also highlights the capability of the LoRa band to penetrate through the deck and walls and the possibility of transmitting IoT data back to the gateway on the top deck. In theoretical calculations, to cope with the uncertainty brought about by the complex environment of ships, an additional 10–15 dB of link budget margin is added above the required minimum receiving sensitivity.

### 3.4. SINR Analysis

**Figure 8:**
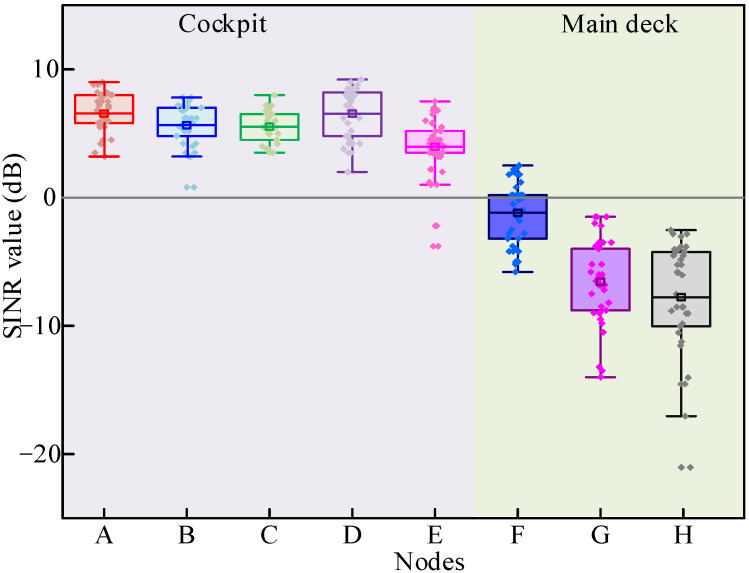
The SINR for both the cockpit and the main deck.

Figure 8 shows the boxplot for the SINR value acquired by all nodes, which includes the mean, minimum, and maximum values. It can be seen that the SINR value acquired by nodes located in the cockpit is almost over 0 dB, which indicates that the signal can be successfully received in the cockpit. Moreover, the SINR values acquired by most nodes located in the cockpit are higher than those of nodes located on the main deck.

Table 6 shows the mean and SD values of SINR acquired by all measurement nodes. Calculation equations of the mean and SD values of SINR are similar to that of RSS. As observed in Table 6, the average SINR value acquired in the cockpit ranges from 4.45 dB to 6.92 dB, while the average SINR value acquired in the main deck ranges from −6.15 dB to −0.59 dB. It can also be found that the SINR values of node F are 5 dB higher than those of nodes G and H. This indicates that the placement of the nodes in the lower deck significantly determines the link quality in the lower deck of the vessel cabin.

Since SINR is a ratio, its performance is directly related to the dB value. Calculating SD at the dB scale is necessary to reflect the fluctuation range of its impact on system performance. As shown in Table 6, nodes G and H located on the main deck have significantly higher SD in SINR (3.26 dB and 4.46 dB, respectively) compared to other nodes. This indicates that the link quality of nodes G and H is in an extremely unstable state.

Nodes G and H are positioned on the main deck at the maximum distance from the gateway, on opposite sides of it, resulting in a critically low RSS. On such a low signal-to-noise ratio basis, the signal is highly susceptible to slight environmental disturbances, leading to significant fluctuations in SINR values. According to the cabin layout, nodes G and H are located in an area with an exceptionally complex electromagnetic propagation environment. When the ship sways slightly or personnel inside the cabin move, the phase relationship of these multipath signals will undergo drastic changes, resulting in serious constructive and destructive interference of the signals, leading to the phenomenon of “deep fading” and “high peak” alternating in SINR, manifested as a huge SD.

Although the absolute signal strength of node C is not the best, its reliability may be the highest, which is a very valuable feature for low-power IoT applications that require stable connections. When deploying networks in complex environments such as ships, it is not only necessary to find the point with the strongest signal, but also to find the point with the most stable signal.

### 3.5. Discussion

**Figure 9:**
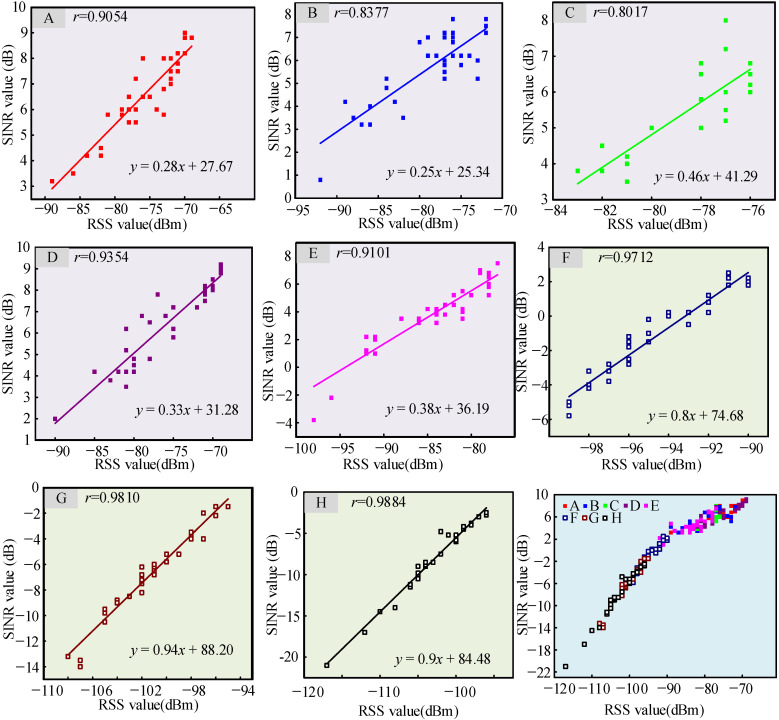
Relationship between SINR and RSS values for nodes **A**–**H**.

Based on data from Figure 7 and Figure 8, the relationship between SINR and RSS values for all measurement nodes is given in Figure 9.

It can be concluded from Figure 9 that there is a linear relationship between SINR and RSS value for most measurement nodes. Moreover, the Pearson correlation coefficient *r* between the SINR and RSS value of almost all nodes was more than 0.8, indicating that the SINR value of most nodes has a positive relationship with the RSS value. That is to say, the noise and interference levels inside the research vessel were stable in the LoRa bands.

The node deployment in this measurement campaign was designed primarily to access the impact of spatial positioning (e.g., side, opposite side, near windows) rather than to model path loss over extended continuous distances. Consequently, the distance variation within each region (bridge and main deck) is constrained, preventing the accurate fitting of a distinct path loss exponent for individual areas. However, when arranging data points from both regions into a larger dataset (spanning 2.5 m to 5.2 m), applying a standard path loss model for validation remains informative. Future work should incorporate measurements at longer distances to enable more robust model calibration.

The RSS values at each measurement location demonstrate significant temporal variability, primarily due to the dynamic nature of the shipboard environment:

▪ Mechanical vibrations: Slight shaking and oscillations of the vessel in the harbor introduce temporal fluctuations in signal strength.
▪ Multipath propagation: The metallic cabin structure generates complex multipath effects, where minor environmental changes (e.g., personnel movement) cause constructive or destructive interference.

While continuous background interference monitoring via a spectrum analyzer was not performed during the experiment, the strong linear correlation between RSS and SINR (Pearson coefficient > 0.8) suggested relatively stable noise and interference throughout the measurement period. This indicates that RSS fluctuations are predominantly driven by channel fading effects rather than abrupt interference variations.

Furthermore, the most promising finding from our measurement campaign is that LoRa modulation combined with the LoRaWAN protocol demonstrated significant potential for enabling reliable marine vessel networks. Notably, the observed signal improvement of 6–9 dB near windows was measured in a port environment, where numerous reflective structures (e.g., other vessels, port infrastructure) likely contributed to this enhancement through constructive multipath propagation.

In contrast, if the vessel operates in isolation on the open sea (where such reflectors are absent), the relative advantage of window proximity is expected to persist due to reduced signal penetration loss through the hull. However, the magnitude of this improvement may diminish compared to port conditions, as multipath effects would be minimized. These observations suggest a valuable direction for future research: validating the reported signal behavior under purely open-sea conditions to assess the robustness of LoRa-based maritime communication systems.

## 4. Conclusions

A radio channel measurement campaign encompassing the 470–510 MHz frequency range was successfully performed. The RSS and the SINR values of measurement nodes were collected and analyzed. The placement of the nodes within two different decks of the top deck (gateway location) was also investigated. The conclusions are as follows:

1. The noise and interference level (electromagnetic environment) remained stable in different decks of the research vessel during the test, whereas small SD in RSS and SINR were caused by the vessel’s movement and the complex cabin environment with multiple paths.
2. The mean RSS values of all measurement nodes exceed the receiver sensitivity (−110 dBm), suggesting that transmitting IoT data in the vessel’s cabin for at least two lower levels is possible and that there is significant potential for signal penetration in LoRa bands in this situation.
3. When deploying the network, it is recommended that the receiving nodes be placed on the same side as the gateway to reduce signal propagation attenuation.
4. The thickness of the steel cabin panels must be considered, and when it exceeds two layers of steel plates, repeaters may be necessary for the network system.
5. Windows may provide additional channels, so they must be considered during deployment.

The SINR values in this study were obtained directly from the LoRa gateway’s physical layer chipset estimation during active data transmission. The interference levels, and consequently the SINR, are expected to be highly dependent on the operational environment. The port environment likely presented a higher interference background compared to the open sea. Therefore, while our results demonstrate the feasibility of LoRa communication within the vessel’s structure in a typical port scenario, the specific SINR values and the potential for even more robust links in open-sea conditions should be considered in future deployments. Generalizing the absolute SINR performance from the port scenario directly to open-sea scenarios should be done with caution, although the relative trends are expected to hold. The node layout in this measurement activity is primarily aimed at evaluating the impact of spatial position, rather than modeling path loss over a continuous long-distance range. When considering the data points of the two regions together as a larger dataset ranging from 2.5 m to 5.2 m, using a standard model for validation still has reference value. Future work should include measurements at longer distances for more reliable model fitting. Additionally, the limited number of sample points constitutes a key limitation of this study. Future work should incorporate a larger number of sampling points and test a wider range of sampling environments to enhance robustness.

## Figures and Tables

**Table 1:** Experimental parameters.

Parameters	Value
Gateway antenna gain	3 dBi
Node antenna gain	3 dBi
Transmission power	22 dBm
Frequency range	470~510 MHz
Measurement logging interval	10 s
SF	12
BW	125 kHz
Permissible transmitting power	0.16 W
Sensitivity of the receivers	−140 dBm
Code rate	4/5
Mode	Class A

**Table 2:** Items of the RXPK JSON object.

RXPK JSON Object	Meaning
Chan	Channel number
rfch	RF link number
Freq	Frequency Of the received data packet
mid	Message ID
stat	Status code
modu	Modulation method
datr	Data rate and bandwidth (i.e., SF and BW)
codr	Coding rate
rssis	Received signal strength measured by the end device
lsnr	Signal-to-noise ratio measured by the end device
foff	Frequency offset
size	Size of the data packet
data	Content of the data packet

**Table 3:** Logarithmic distance path loss model prediction values.

Locations	d (m)	PL_Measured_ (dB)	PL_predict_ (dB)	Residual (dB)
A	2.5	105.80	104.53	1.27
B	3.5	108.62	115.75	−7.13
C	3.3	109.99	113.79	−3.80
D	2.5	104.97	104.53	0.44
E	2.8	113.70	108.31	5.39
F	5.1	125.73	128.31	−2.58
G	5.2	131.81	128.96	2.85
H	5.2	132.49	128.96	3.53

**Table 4:** InF-SL model prediction values.

Locations	d (m)	PL_Measured_ (dB)	PL_InF-Calculated_ (dB)	Residual (dB)
A	2.5	105.80	106.12	−0.32
B	3.5	108.62	118.23	−9.61
C	3.3	109.99	116.11	−6.12
D	2.5	104.97	106.12	−1.15
E	2.8	113.70	110.20	3.5
F	5.1	125.73	131.79	−6.06
G	5.2	131.81	132.49	−0.68
H	5.2	132.49	132.49	0

**Table 5:** Mean value and SD of RSS.

Location	Node	Mean Value of RSS (dBm)	SD of RSS (dB)
Cockpit	A	−73.79	5.00
	B	−76.61	5.40
	C	−77.99	2.15
	D	−72.97	5.53
	E	−81.70	5.41
Main deck	F	−93.73	2.88
	G	−99.81	3.42
	H	−100.49	4.90

**Table 6:** Mean value and SD of SINR.

Location	Node	Mean Value of SINR (dB)	SD of SINR (dB)
Cockpit	A	6.81	1.54
	B	5.91	1.61
	C	5.68	1.23
	D	6.92	1.94
	E	4.45	2.28
Main deck	F	−0.59	2.38
	G	−5.50	3.26
	H	−6.15	4.46

## Data Availability

The original contributions presented in this study are included in the article. Further inquiries can be directed to the corresponding author.
